# Failed prey or peculiar necrolysis? Isolated ammonite soft body from the Late Jurassic of Eichstätt (Germany) with complete digestive tract and male reproductive organs

**DOI:** 10.1186/s13358-020-00215-7

**Published:** 2021-01-18

**Authors:** Christian Klug, Günter Schweigert, Helmut Tischlinger, Helmut Pochmann

**Affiliations:** 1 Paläontologisches Institut Und Museum, Universität Zürich, Karl-Schmid-Strasse 4, 8006 Zurich, Switzerland; 2 Staatliches Museum Für Naturkunde, Rosenstein 1, 70191 Stuttgart, Germany; 3 Tannenweg 16, 85134 Stammham, Germany; 4 Jura-Museum Eichstätt, Willibaldsburg, 85072 Eichstätt, Germany; 5 Königsdorfer Straße 24, 82547 Eurasburg, Beuerberg, Germany

**Keywords:** Cephalopoda, Ammonoidea, Pycnodontiformes, Coleoidea, Tithonian, Predation, Taphonomy, Conservation deposits, Dimorphism, Anatomy

## Abstract

Ammonoid soft parts have been rarely described. Here, we document the soft parts of a perisphinctid ammonite from the early Tithonian of Wintershof near Eichstätt (Germany). This exceptional preservation was enabled by the special depositional conditions in the marine basins of the Solnhofen Archipelago. Here, we document this find and attempt to homologize its parts with various organs such as the digestive tract, reproductive organs, the mantle cavity with gills, and the hyponome, with differing degrees of reservation. Alternative interpretations are also taken into account. We suggest that the soft parts were separated from the conch either taphonomically (following necrolytical processes affecting the attachment structures) or during a failed predation, where a predator (fish or coleoid) removed the soft parts from the conch but then dropped them. This find is interesting because it adds to the knowledge of ammonite anatomy, which is normally hidden in the conch. The reproductive organs show traces of what might have been spermatophores, thus supporting the hypothesis that the microconchs represented the males.

## Introduction

Ammonoid conchs and jaws are known in great detail from a plethora of publications, while reports of identifiable soft parts are exceedingly rare or doubtful (Kolb 1961, 1967; Closs 1967a, b; Zeiss 1968, 1969; Stürmer 1969; Otto 1994; Hollingworth and Hilton 1999; Schweigert and Dietl 1999; Klug and Lehmann 2015; Lehmann et al. 2015; Klug et al. 2019; Donovan and Fuchs 2016; Clements et al. 2016; Mapes et al. 2019). To some degree, this can be explained by the fact that the soft parts are surrounded by the conch and thus, even if they are preserved, they are hidden under the shell. Also, sclerotized parts are rare in their bodies and limited to jaws, radula and the oesophagus.

Ammonoid soft-part preservation requires depositional conditions as found in conservation deposits, although the specimen published by Hollingworth and Hilton (1999; see also Klug and Lehmann 2015) shows that unusual preservation might also occur in unexpected sedimentological contexts.

Most ammonoid materials preserving remains of the soft parts come from conservation deposits worldwide. These include specimens from the Late Devonian of Morocco (Klug et al. 2016a, b), the Early Carboniferous of Bear Gulch (Landman et al. 2010; Klug et al. 2019; Mapes et al. 2019), the Late Carboniferous of Paraguay (Closs 1967a, b; Bandel 1988; Lehmann et al. 2015), the Permian of the USA (Tanabe et al. 2000); the Early Triassic of Greenland (Lehmann 1985), the Middle Triassic of Germany (Klug and Jerjen 2012), the Late Triassic of Austria (Doguzhaeva et al. 2004), the Early Jurassic of Germany (Lehmann and Weitschat 1973; Wetzel 1979; Lehmann 1985; Riegraf et al. 1984) and Great Britain (Lehmann and Weitschat 1973), the Middle Jurassic of Russia (Mironenko 2015a), the Late Jurassic of Germany (Schweigert and Dietl 1999; Mapes et al. 2019), as well as the Late Cretaceous of Lebanon (Wippich and Lehmann 2004) and of Germany (Klug et al. 2012, 2015).

Here, we describe ammonite remains from Late Jurassic conservation deposits of Nusplingen and Wintershof (southern Germany), which are only rarely preserved otherwise. Both localities are known for their Late Jurassic platy limestones, which yield sometimes exceptionally preserved cephalopod fossils (Schweigert and Dietl 1999; Dietl and Schweigert 2001; Klug et al. 2005, 2010a, b, 2015, 2016a, b; Fuchs 2006a, 2015; Keupp et al. 2011; Arratia et al. 2015; Meyer 2015; Schweigert et al. 2016). In the region of Wintershof, these limestones were laid down during the early Tithonian (Hybonotum Zone, Riedense Subzone, *eigeltingense* β Biohorizon), while the limestones of Nusplingen are a little bit older and of late Kimmeridgian age (Beckeri Zone, Ulmense Subzone) (Schweigert 1998, 2007).

Remarkably, the ammonite remains portrayed in this study are buried outside their conchs. Here, we will first describe these remains, secondly attempt a homologization of the single parts, and thirdly discuss the taphonomic history.

## Material and methods

The studied specimens come from the two localities Nusplingen (late Kimmeridgian; see Dietl and Schweigert 2001 for locality information) and Wintershof (early Tithonian; see Arratia et al. 2015 for locality information). The main specimen, which was discovered by HP, comprises jaws and soft parts of an ammonite from the Tithonian of Wintershof. This specimen and a second specimen from Nusplingen are stored at the Staatliches Museum für Naturkunde in Stuttgart (SMNS-numbers). A further specimen, which was collected by Franz-Xaver Schöpfel, comprises the conch with the aptychus in situ from Wintershof (Meyer 2015). It is kept in the Bayerische Staatssammlung für Paläontologie und Geologie in Munich (SNSB-BSPG 2014 XXI 79392).

**Fig. 1:**
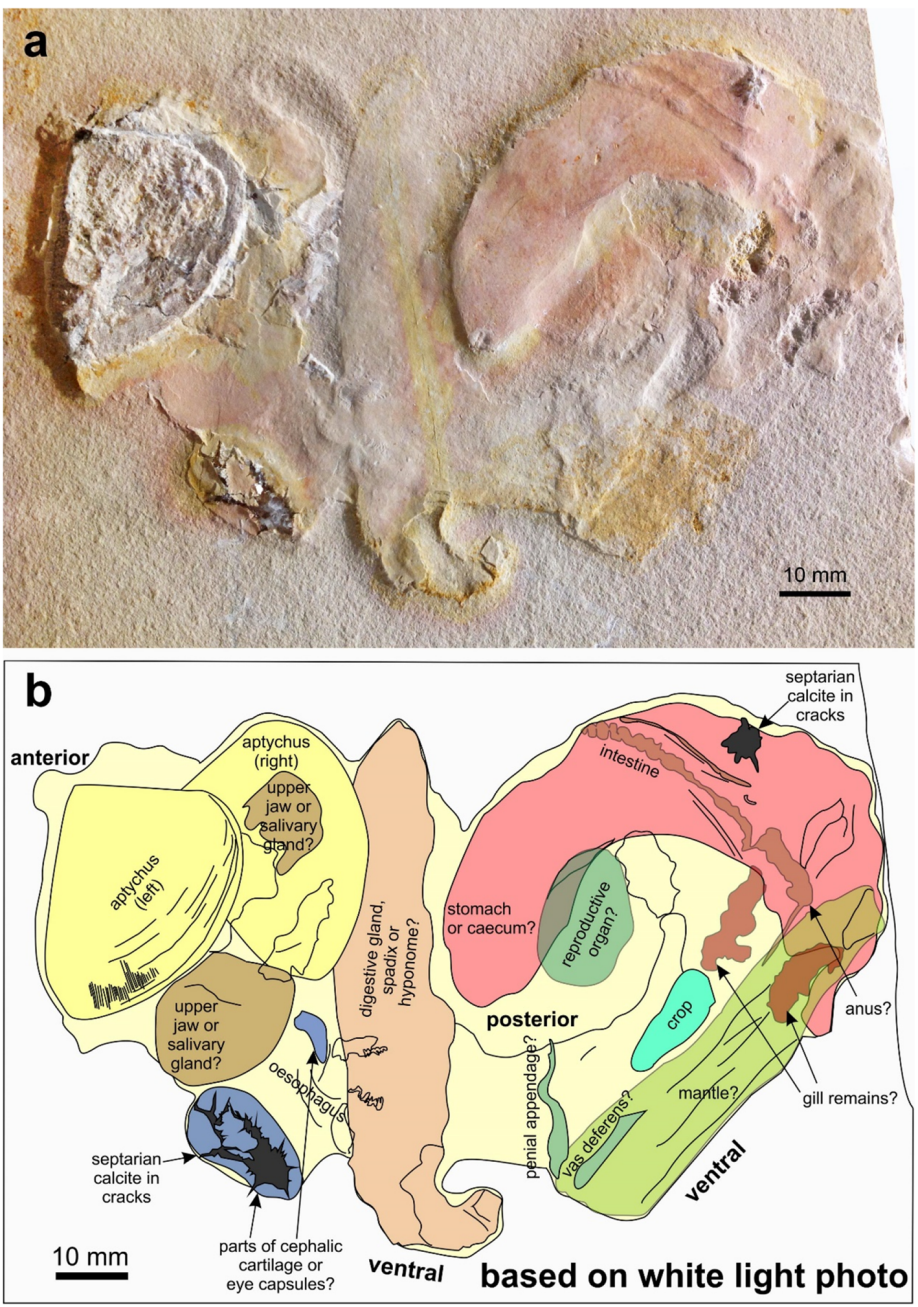
Soft parts of *Subplanites* sp. with a *Strigogranulaptychus* sp. from the early Tithonian of Wintershof near Eichstätt (Germany); SMNS 70,610. **a** Photo taken under white light. **b** Line drawing of the structures visible in the white light-photo (**a**) with possible interpretations

**Fig. 2:**
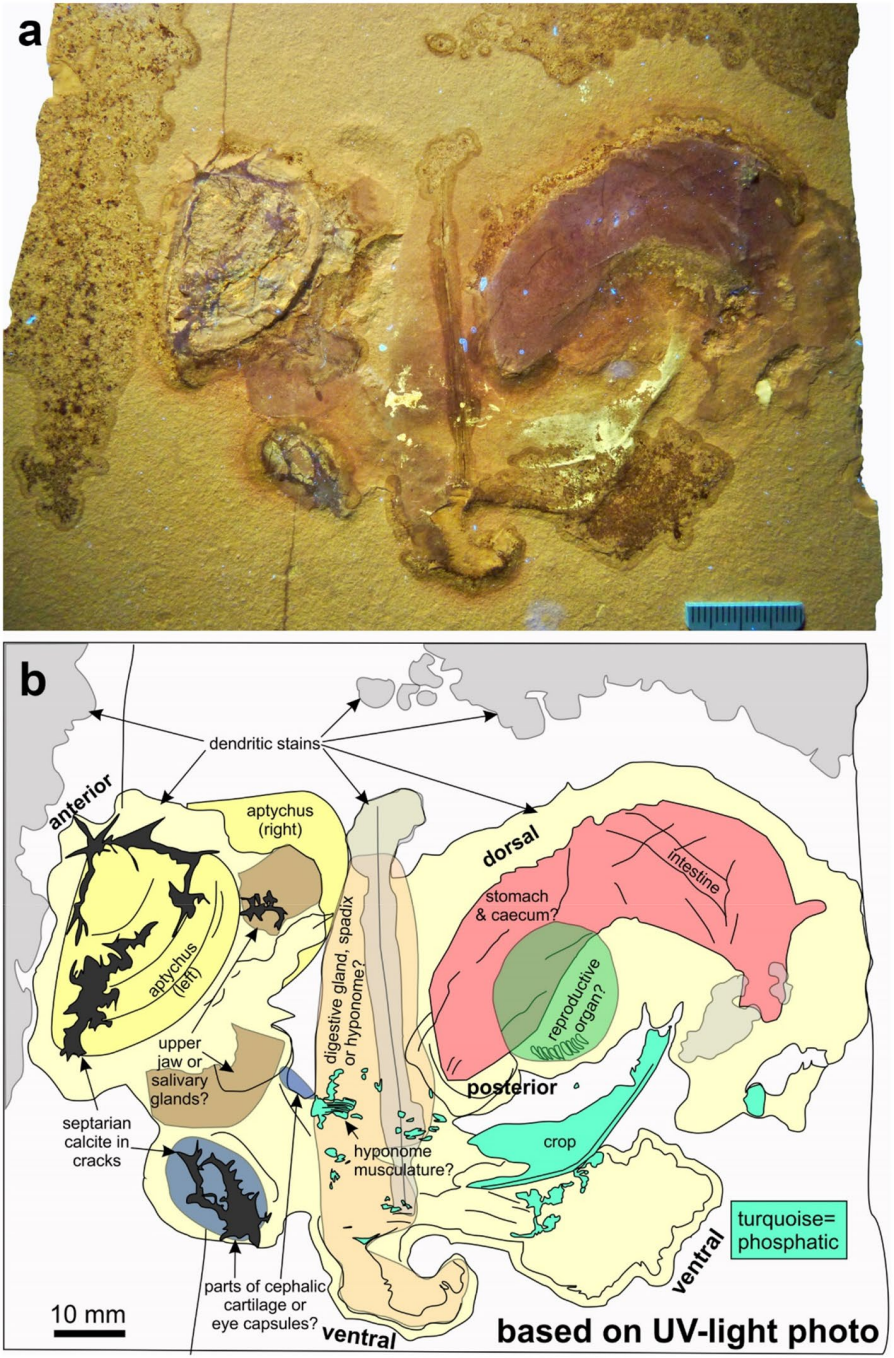
Soft parts of *Subplanites* sp. with a *Strigogranulaptychus* sp. from the early Tithonian of Wintershof near Eichstätt (Germany); SMNS 70610. **a** Photo taken under UV-light. **b** Line drawing of the structures visible in the UV-photo (**a**) with possible interpretations

We photographed the main specimen SMNS 70610 using white light (Fig. 1a) and UV-light (Fig. 2a), because the latter helps to make faintly phosphatized structures better visible (Tischlinger and Arratia 2013; Tischlinger 2015). The UV documentation was carried out with the help of a high-performance Labino UV-A lamp, Spotlight S 135, 35 W, 365 nm, equipped with a custom-made midlight-reflector-inset.

## Results

### Description

The main specimen SMNS 70610 is a limestone plate with remains of an ammonite (Figs. 1, 2). We assign these remains to the genus *Subplanites* based on the presence of a *Strigogranulaptychus*, the early Tithonian age of the sediments in the quarry at Wintershof, and the abundance of representatives of this genus at that locality. The plate measures 175 × 165 mm. Of this surface, the fossil remains occupy 132 × 82 mm. These remains are coiled with the coil running from the aptychus down towards the centre, then upwards to the right and turning back to the left upwards.

We describe the specimen starting from the buccal mass to the opposite side, i.e. starting at the mouth and then follow the course of the digestive tract. The *Strigogranulaptychus* is preserved in calcite, 37 mm long and 28 mm wide (yellow in Figs. 1b and 2b). Only one half of the lower jaw is visible. The UV-photo revealed that septaria-like calcite veins cross the jaw in some places (Fig. 2). It displays concentric growth lines and a very fine radial striation. The other half is not visible, but a bulge above and to the right of the visible aptychus is here interpreted as bearing the second aptychus below.

Also on the right side of the aptychus, there are two rounded fields coated with a thin layer of an iron oxide (probably haematite; brown in Figs. 1b and 2b). Below the aptychus, there is an oval structure with septarian calcite veins (blue in Figs. 1b and 2b). It measures about 21 × 12 mm. Above it, two concave furrows are visible and a 8-mm-long kidney-shaped structure (blue in Figs. 1b and 2b).

Further to the right, an elongate structure measuring 83 × 18 mm follows (beige in Figs. 1b and 2b). Its left margin is distinct and slightly curved, while the right margin is more irregular. The UV-photo (Fig. 2b) revealed some patches of striated phosphate. The appearance of these patches is reminiscent of the mantle musculature of coleoid mantle from Solnhofen (cf. Klug et al. 2016a, b). At the lower end of this structure, it appears slightly coiled, but this could as well be a taphonomic artefact.

On the top right, another distinct structure follows, which is elongate and curved, thus nearly kidney-shaped (pink in Figs. 1b and 2b). It extends over 68 × 47 mm and is covered by a fine film of iron oxides (probably haematite because of the colour). Its left end points downward, is somewhat pointed and has a little notch at the end. Over most of its length, it is about 17 mm wide. On the right end, its limit is irregular and less distinct. Towards the top of this structure, some smaller structures are discernible. At the top, there is again a small septarian calcite, which might be of purely diagenetic origin (dark grey in Figs. 1b and 2b). The second structure is elongate and obliquely crosses the pink structure from the top right to the lower left (light brown in Fig. 1b). It consists of a 32-mm-long and 3-mm-wide elevation with small irregular bulges and becomes thinner towards the left. Further to the left, it is followed by a 13-mm-long and 3-mm-wide structure with an irregular surface that shares the same orientation. This is attached to a furrow, which is oriented perpendicularly to the lower left.

**Fig. 3:**
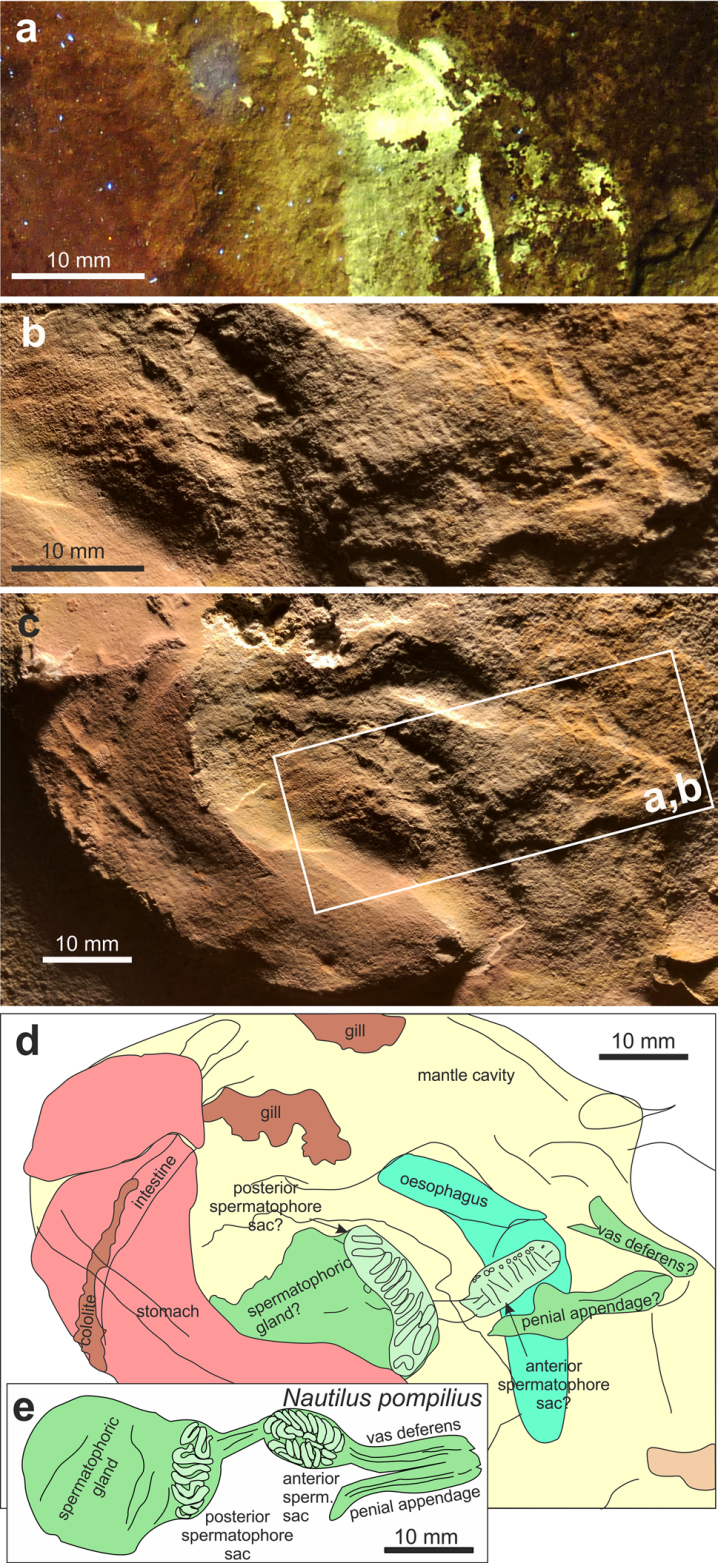
Detail of the soft parts of *Subplanites* sp. to show details of the reproductive organs (green colours). **a** Detail of the photo in b taken with UV-light; the fluorescent structure is part of the crop. **b** Like **a**, with white light. **c** Photo taken under shallow white light to show the remains of what might be reproductive organs. **d** Line drawing of the structures visible in the b with possible interpretations. **e** Drawing of the male reproductive organs of *Nautilus pompilius* (after Sasaki et al. 2010: Fig. 12E)

Below the pink structure, there is an oval element, which measures 25 × 15 mm (green in Figs. 1b and 2b). Under UV-light, it shows a pattern of small, comma-shaped elevations at its lower margin (Fig. 2b, 3). There are about ten such elevations, which are ca. 1 mm wide and 2 to 4 mm long. They are slightly curved and more or less parallel to each other. In a close-up photographed under white light, further details became visible. Next to the oesophagus lies a striated structure. Anterior to it, two elongate furrows measuring about 15 and 20 mm in length can be seen; they are arranged at an acute angle (green structures on the right in Fig. 3c).

Under UV-light, a boomerang-shaped object became visible, which is likely phosphatized and barely visible under white light (turquoise in Fig. 2b). The fluorescent structure is 34 mm long and appears to have two lappets at the left end, which are nearly 9 mm high. It has a kink at the middle and a thickly phosphatized lower edge. At both ends, the phosphate coating wedges out. Under white light, there is only an elongate suboval furrow (turquoise in Fig. 1b).

At the bottom right, there is a subrectangular structure, which varies in width between 15 and nearly 20 mm (green in Figs. 1b and 2b). It is roughly 65 mm long and carries some longitudinal folds. At its top right end, there are two nearly symmetrical depressions, which are 15 and 16 mm long and 5 mm wide (brown in Figs. 1b and 2b). They both share an irregular outline and a rough surface. Their long axes run in parallel to each other and to the longer structure described just before. Under UV-light, the lower one of the two depressions contains a small spot with phosphate.

## Discussion

### Homologization of organs

**Fig. 4:**
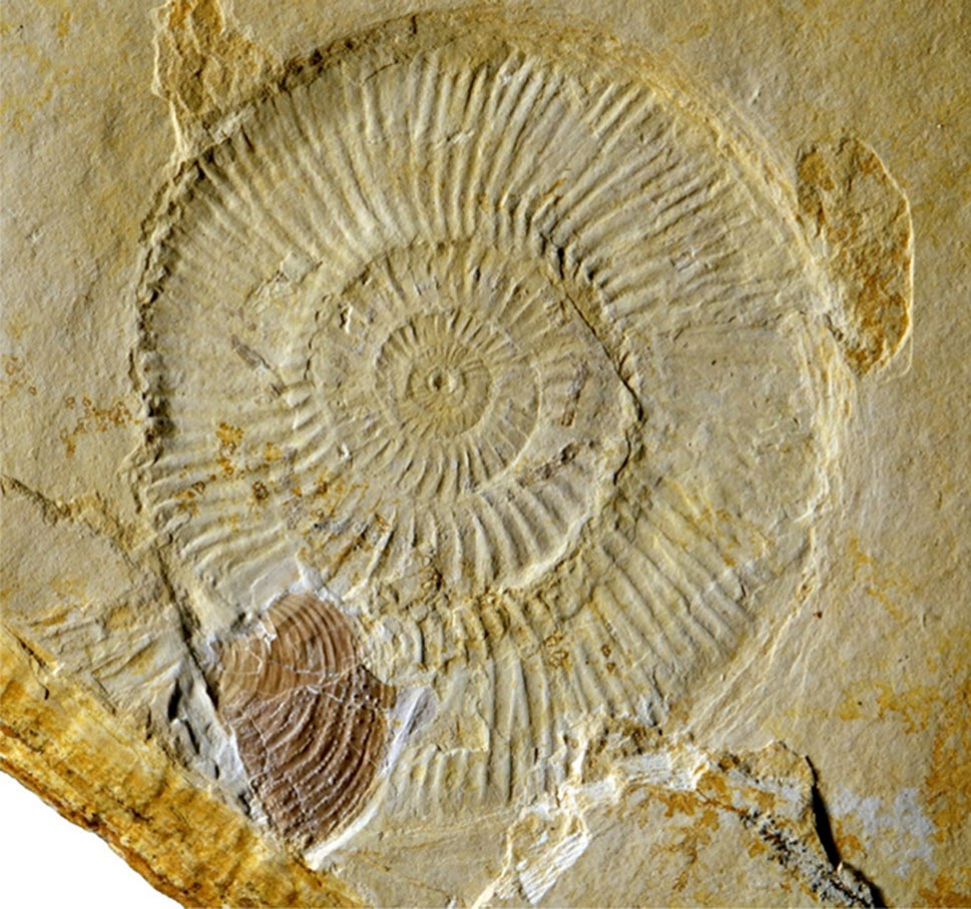
Complete conch of the perisphinctid ammonite *Subplanites* sp. with its *Strigogranulaptychus* sp. from the early Tithonian of Wintershof near Eichstätt (Germany); conch diameter 90 mm; SNSB-BSPG 2014 XXI 79392. Photo reproduced from Keupp and Schweigert (2015: Fig. 415) with kind permission of F. Pfeil (publisher) and the authors

The presence of the aptychus corroborates the ammonite-affinity of the fossil and provides an orientation. Normally, the buccal mass is located anterior to the digestive tract, mantle cavity, brain, reproductive organs and other internal organs. We suggest that the coiling of the structures to the right of the jaw in Figs. 1 and 2 followed the coiling of the body chamber. The aptychus morphology suggests an affinity of the ammonite to the genus *Subplanites*, which is well known from the same locality and strata as SMNS 70610. The *Subplanites* depicted in Fig. 4 (SNSB-BSPG 2014 XXI 79392) preserves the aptychus and the phosphatized siphuncle, although the latter is slightly fragmented. In this specimen, the distribution of siphuncular fragments suggests that the body chamber occupied about 300° of the last whorl, which fits well with the coiling mode and conch geometry of this ammonite (cf. Westermann 1996; Korn and Klug 2003; Klug and Korn 2004). It also coincides with a roughly horizontal orientation of the aperture during life.

Independent of the question how the soft parts became separated from the conch, we assume that they sank to the sediment from somewhere in the water column: there are no signs of trace fossils surrounding the soft parts, which should be present if predation or scavenging occurred on the sediment (e.g., Vallon et al. 2015). A fast separation of soft parts was documented for nautilids (Wani et al. 2005; Wani, 2007). It is also likely that the aptychi touched the sediment first since they are the heaviest part within the body chamber. The respective lengths of dorsal and ventral tissues to some extent retained the coiling of the body chamber.

Presuming that the assumption about the coiling of the soft parts reflecting the body chamber coiling is correct, we hypothesize that the organs are arranged in a manner typical of cephalopods. Soft tissue anatomy of ammonites is poorly known (Klug et al. 2015 and references therein). Nevertheless, the aspects of the internal anatomy that are known from ammonites (Lehmann and Weitschat 1973; Lehmann 1981, 1985; Landman et al. 2010; Klug et al. 2012; Lehmann et al. 2015) and early coleoids (Landman and Davis 1988; Fuchs 2006a, b; Klug et al. 2019) suggest a rather simple arrangement being similar to that in, e.g., modern nautilids (Ward 1987; Sasaki et al. 2010). These thoughts are important for the homology criterion of position in relation to other organs and also for the criterion of evolutionary continuity. Subsequently, we will discuss these and add morphological details as far as they are discernible as arguments for the criterion of specific quality and structure.

**Fig. 5:**
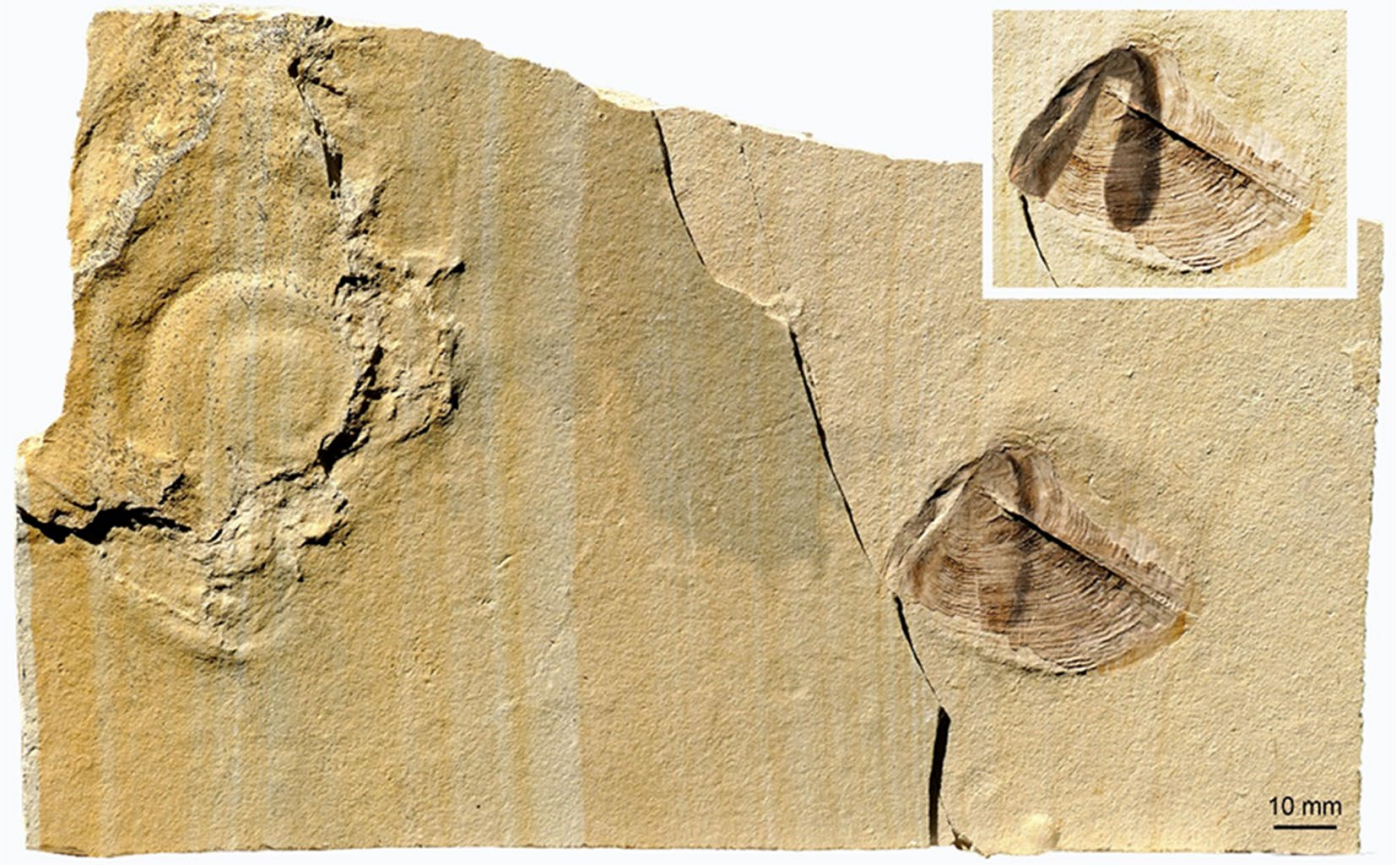
Fragmentary conch of a perisphinctid with complete jaw apparatus (*Praestriaptychus*) next to it from the late Kimmeridgian of Nusplingen (SMNS 70613). The facts that upper and lower jaws are still in contact (insert with modified brightness to show the arrangement of the upper jaw), that the buccal mass is so close to the damaged conch that all parts likely belonged to the same species, and the dark stain between these parts suggest that they were parts of the same individual

The localization of the buccal mass is corroborated by the presence of one well-preserved aptychus. The second part might be present slightly below, still covered by soft parts and sediment (Figs. 1, 2). Below and right of the aptychus, slightly haematitized surfaces document the presence of an organ that was either non-sclerotized or poorly sclerotized. The two structures (brown in Figs. 1b and 2b, adjacent to the aptychus) could be the wings of the upper jaw. The upper jaw is usually chitinous and not mineralized in ammonite jaws (Kruta et al. 2011; Tanabe et al. 2015). It tends to have elongate wings in perisphinctids (Fig. 5). Since some faintly chitinous structures such as coleoid beaks are poorly recognizable in the Late Jurassic of the Eichstätt region, these two patches might represent the posterior ends of the wings of the upper jaw. However, there is no structure providing hard evidence for this interpretation. The buccal mass is associated with salivary glands, so it is conceivable that these patches might alternatively represent remains of this gland.

Posteriorly, the buccal mass is followed by the oesophagus and crop in many cephalopods including nautilids and some coleoids. In ammonoids, oesophagus and crop usually have a chitinous sheet and thus have some potential to be fossilized (Klug and Jerjen 2012; Klug et al. 2012). The oesophagus is in many cephalopods rather short and surrounded by the brain (Nixon and Young 2003). Hence, its homologization is linked with the identification of brain and eye capsules. In Figs. 1b and 2b, some structures follow below the aptychus on the right, which are coloured in blue. The big oval could represent an eye capsule, although specific anatomical details are not visible. Between the spots marked in blue, a furrow is visible, which could well be an imprint of the oesophagus. Presuming the eyes were really positioned there, this raises questions for the position of jaws and eyes when the ammonite was active: did the entire buccal mass extend out of the aperture or could the eyes move to the sides of the buccal mass?

Remarkably, the crop appears to differ between forms with straight conchs (baculitids: Klug et al. 2012, 2015) and coiled conchs (nautilids: Moore 1964; Sasaki et al. 2010; ceratitids: Klug and Jerjen 2012). In the baculitids, the crop appears to be straight, while both in nautilids and coiled ammonoids, the crop shows a kink (Moore 1964; Sasaki et al. 2010; Klug and Jerjen 2012) and is composed of two parallel elongate bulges in the anterior part in ceratitids (Klug and Jerjen 2012: Fig. 4a). The phosphatized structure that appears whitish in Fig. 2a and is marked in turquoise in Fig. 2b shows exactly this kink and the two anterior lobes. The fact that it is phosphatized highlights its possibly originally chitinous cover. Accordingly, all main homology criteria (position, specific quality, continuity) are fulfilled to confidently identify this structure as crop. Alternatively, it could perhaps be the cephalic retractor muscle.

Above the crop, a large, kidney-shaped structure is visible, which shows a fine cover of reddish iron oxides (red in Figs. 1b and 2b). It lacks smaller structures directly supporting its homologization with an organ. In this context, it appears reasonable to discuss the elongate structure marked in brown in Figs. 1b and 2b near the top of the structure marked in red. It apparently lies below the large reddish structure. The elongate structure shares some characters such as its dimensions, its elongate and slender form, and the irregular constrictions with the ichnogenus *Lumbricaria* (Janicke 1970; Kietzmann and Bressan 2019; Knaust and Hoffmann 2020). Accordingly, we suggest that this is a cololite and the structure corresponds to the intestine. The intestine, in turn, originates from the stomach. Consequently, we suggest that the large reddish structure represents the stomach, maybe overlapping the caecum.

The stomach partially covers an oval structure, which is marked in green in Figs. 1b and 2b. It shows a serial pattern at its lower right edge. Its position suggests that it was originally located in the posterior part of the body chamber. In modern *Nautilus*, this part hosts the reproductive organs (Sasaki et al. 2010). The ovaries appear to have a rather smooth surface, while the spermatophores correspond in dimensions and arrangement to the regular pattern seen in this organ. Fossil cephalopod spermatophores have been reported from Painten, which is not too far away from the Eichstätt region and only slightly older than the ammonite described here (Keupp et al. 2011). With some reservation, we thus suggest that it might represent the spermatophoric gland with the anterior and posterior spermatophore sac with about ten visible spermatophores each as well as the vas deferens and penial appendage (Fig. 3b, c). The position and proportions (including the dimensions of spermatophores) of the fossilized structures conform well to those of the parts of the reproductive organs of *Nautilus pompilius* (Fig. 3e) as depicted by Sasaki et al. (2010: Fig. 12E). Additionally, the interpretation as male organs coincides with conch size and the determination a *Subplanites* (macroconch counterpart might be *Euvirgalithacoceras*: Zeiss et al. 1996).

Below stomach and reproductive organs, a large elongate organ is marked in green in Figs. 1b and 2b comprising the two elongate depressions marked in brown at its upper right end. Presuming the elongate structure coloured in brown in Fig. 1b near the top of the stomach is indeed the intestine, the intestine would terminate near the posterior end of the structure marked in green between the paired depressions marked in brown. This suggests an interpretation that the green structure may be the part of the mantle surrounding the mantle cavity.

In all cephalopods, the gills are attached to the posterior part of the mantle cavity (Lehmann and Weitschat 1973; Lehmann 1981, 1985; Reitner 2009; Sasaki et al. 2010; Klug et al. 2015, 2019; Mironenko 2015a). From ammonites, only very few good records of gill remains are available from the Mesozoic (Lehmann and Weitschat 1973; Lehmann 1985; Mironenko 2015a). In all cases, the gills are located ventrally in the posterior mantle cavity. In spite of the absence of distinct morphological details, we suggest that the two depressions probably represent imprints of the gills.

**Fig. 6:**
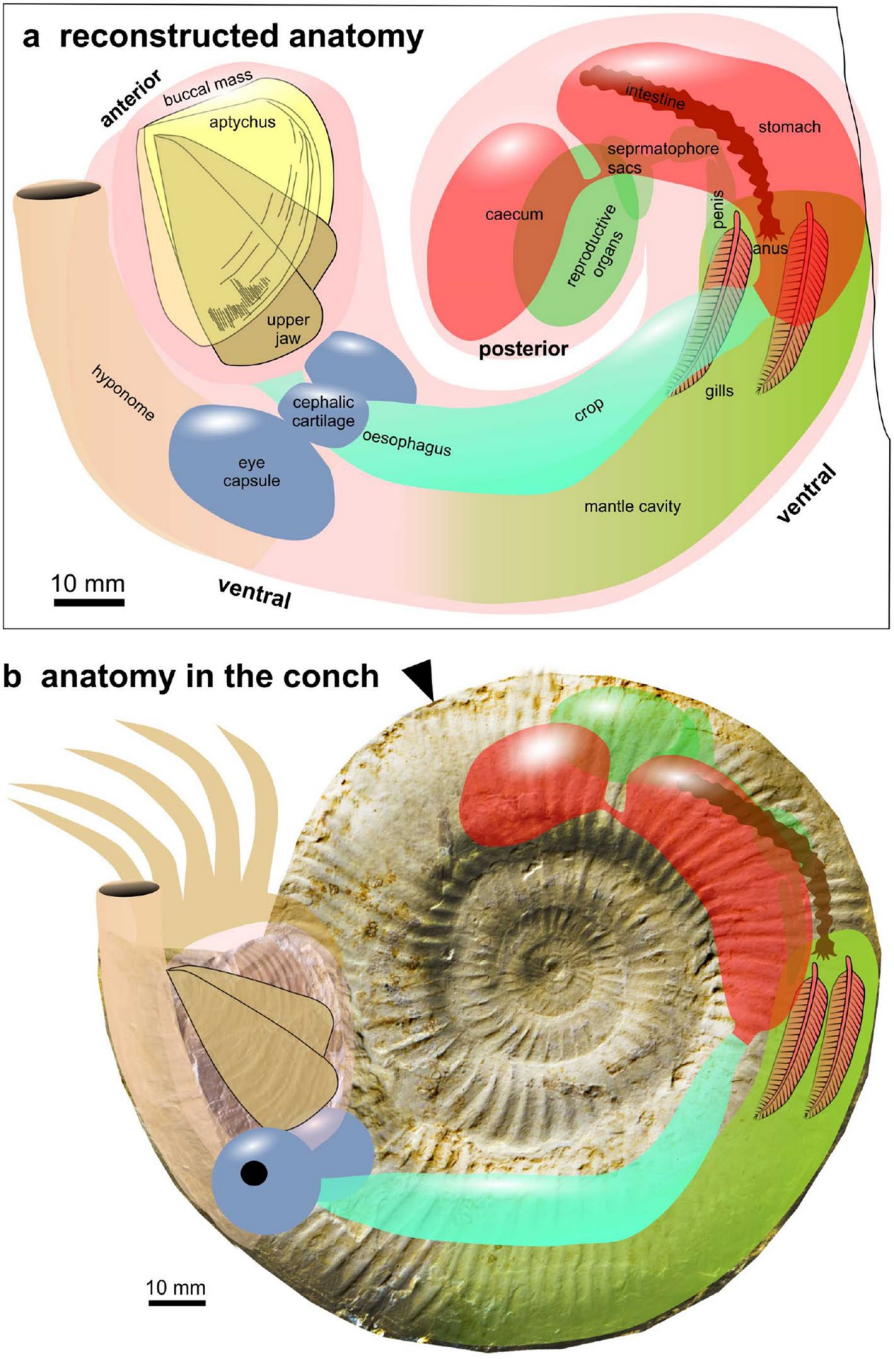
Reconstruction of the internal anatomy of *Subplanites* as it came to rest on the sediment. Note that the interpretations of some organs such as the reproductive organ, the central nervous system, the hyponome and the gills are a matter of debate and represent only one possible interpretation out of several. The coiling of the soft parts corresponds to the coiled body chamber (ca. 300°), which was probably altered when these soft parts sank onto the sediment and came to a rest. **a** Reconstructed as in the fossil. **b** Organs arranged according to the conch in Fig. 3

Between buccal mass and stomach, there is a vertically oriented structure which is nearly straight and of considerable proportions (beige in Figs. 1b and 2b). Within this elongate structure, some phosphatized structures are preserved (marked in turquoise in Fig. 2b). Presuming this is only a relic of more extensive musculature, we infer that this is a formerly muscular structure. Its anterior position and elongate shape is here provisionally interpreted as the hyponome, although it cannot be excluded that this is a digestive gland, a fragment of the mantle, or even a reproductive organ. If our interpretation of the reproductive organs as those of a male animal is correct, this could also be the spadix. These interpretations are summarized in Fig. 6.

Organs we could not identify are the heart, the retractor muscles, and the arms. The arm crown is certainly absent (no fossil structures anterior of the buccal mass), while it is conceivable that the others are present but we were unable to recognize them.

### How were the soft parts separated from the conch?

Several hypotheses are at hand to explain how these ammonite soft parts became separated from the conch: (1) the soft parts were only loosely attached to the conchs as in female *Argonauta* (Conrad 1854; Finn 2009); (2) the soft parts fell out of the shell post mortem, when all tissues attaching them to the shell started to decay (cf. Allison 1988; Wani et al. 2005; Wani 2007; Clements et al. 2016); (3) the soft parts were actively pulled out of the body chamber by a predator.

The first hypotheses (1) appears unlikely because the conch of female argonauts is formed by the arms and not by the mantle (Conrad 1854; Finn 2009), it is not attached by muscles as known from ammonoids (Doguzhaeva and Mapes 2015), and the argonaut conch is not chambered. The attachment of the ammonoid body to the conch (e.g., Doguzhaeva and Mapes 2015) is thus considered much stronger than in argonauts. Also, the argonaut conch is not homologous to the ammonoid conch.

As demonstrated by Clements et al. (2016), decay proceeds quite quickly in cephalopods (cf. Briggs and Wilby 1996). After the death of an ammonite, putrefaction gases may have made the ammonite carcass to float up to the water surface. Depending on the water depth and temperature, sinking might have occurred. Possible scenarios were discussed by several authors (Wani et al. 2005; Wani 2007; Wani and Gupta 2015; Yacobucci 2018). After some weeks at the latest, the attachment of the soft body near the aperture and near the end of the body chamber probably became loose. Nevertheless, as long as the phragmocone contained significant amounts of gas, the body chamber would have been below it, with the aperture facing more or less upward (thick epizoan crusts might have altered this: Donovan 1989; Stilkerich et al. 2017). Also, the mantle sealed off the rear part of the body chamber; in order to pull out the soft parts, the developing space between the last septum and the soft parts had to be filled by water. This likely produced some mechanical resistance against the soft parts falling out of the conch (in localities of the Holzmaden and Solnhofen regions, the jaws are often preserved within the body chambers, thereby corroborating the fact that the soft parts were decaying within the body chamber). Additionally, a strand of soft tissues entered the siphuncle (e.g., Tanabe et al. 2000), further fixing the soft parts to the conch.

Concerning hypothesis (2), we assume that either the soft parts were eaten by scavengers or remained in the body chamber and did not simply fall out of the body chamber due to decay of some tissues. Nevertheless, we cannot rule out that occasionally, the attachments of various tissues behind the aperture and in the posterior part of the body chamber (Mironenko 2015b) became loose during decay and possibly, due to wave action or something else, the soft parts were shaken out of the conch (Keupp and Schweigert 2015).

The third hypothesis (3) is difficult to test. It is striking that the arm crown is missing while traces of many important non-mineralized and non-sclerotized organs are present (independent of the correctness of our attempt of homologizing these organs). Also, specimens such as the ammonite with the separated buccal mass shown in Fig. 4 represent likely documents of predation or scavenging on ammonoids. As cephalopods living in only moderately deep water in great numbers, ammonites probably represented important food sources of several larger predators (e.g., Keupp 2012; Tajika et al. 2018, 2020). A widespread phenomenon are holes in the conch near the posterior end of the body chamber (Klompmaker et al. 2009). This phenomenon is too common to have happened by accident. Klompmaker et al. (2009) already pointed out that this predatorial strategy bears the advantage that this was the ‘blind spot’ of ammonites, where they would possibly overlook a predator. Similarly important, this way, the predator could access all of the soft parts, which were firmly attached by muscles and other tissues at the rear end of the body chamber. In contrast, a predator trying to access the soft parts from the aperture faced two problems: the ammonite could demonstrably withdraw its body deep into the body chamber (Kröger 2002) and at the front end, it was somewhat protected by the calcified lower jaws. Therefore, it appears well conceivable that the soft parts described here were a prey item of a predator capable of breaking the shell, who was aware of the advantage of opening the conch near the rear end of the body chamber. For some reason such as poor visibility or distraction, it dropped its prey and could not retrieve it. The non-preservation of the arm crown may be linked with this predatorial strategy, where it might have been ripped off the rest of the soft parts. It is also conceivable that the arm crown lies in a different plain above or below the fossil so that they are either not visible or not preserved any more. Furthermore, they might have decayed quicker than the rest, although this appears unlikely.

### Which animals fed on ammonites?

It is not possible to unequivocally answer the question for the predator. Since the attack likely happened near the rear end of the body chamber, a certain degree of intelligence can be inferred, pointing at a vertebrate or cephalopod predator. From the Solnhofen Archipelago, many potential vertebrate culprits are known (Frickhinger 1994, 1999; Arratia et al. 2015). One candidate could be members of the Pycnodontiformes, since they have been convincingly shown to have attacked ammonites in the way described above (Richter 2009). Their dentition was strong enough to break ammonite conchs. In addition to the imprints of teeth in the conch characteristic for pycnodont fishes (Martill 1990; Richter 2009), these fishes followed exactly the strategy described by Klompmaker et al. (2009). Nevertheless, it is well conceivable that other vertebrates like other fishes or reptiles like turtles or ichthyosaurs as well as cephalopods also learned how to feed efficiently on ammonites without ingesting the whole conch as well and without missing on much of the nutritious soft parts.

**Fig. 7:**
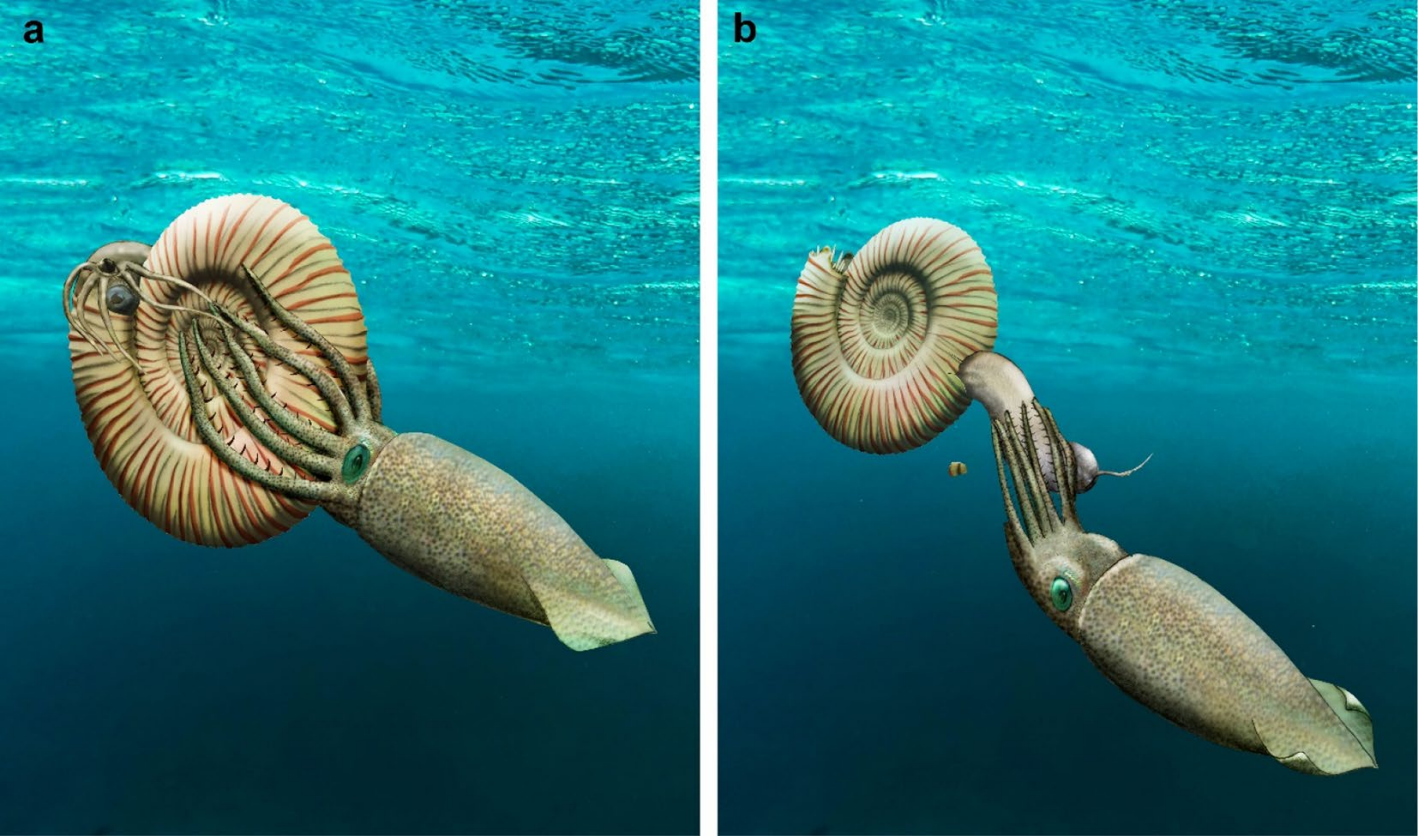
Reconstruction of a possible sequence of events that led to the separation of the soft parts from the conch of *Subplanites.* **a** *Acanthoteuthis* attacks the ammonite and breaks the conch in the posterior half of the body chamber. **b** *Acanthoteuthis* withdraws the soft parts. Possibly, the arm crown remained attached to the aperture or was eaten away otherwise

Among the cephalopods, belemnites like *Hibolithes*, belemnoteuthids like *Acanthoteuthis* as well as some octobrachian coleoids such as *Leptotheuthis, Plesioteuthis* or *Trachyteuthis* are genera that share a sufficiently large body size, strong arms, and well-sclerotized jaws (Clarke 1962, 1986; Clarke and Maddock 1988; Klug et al. 2005, 2010a, 2015, 2016a, b, 2017, 2020; Fuchs 2006a, b; Fuchs and Larson 2011a, b; Tanabe 2012; Jattiot et al. 2015; Keupp and Mitta 2015; Nixon 2015; Jenny et al. 2019). The great abundance of ammonite conchs with a hole in the posterior body chamber is a strong argument for a coleoid predator, since they are much more common in the Late Jurassic conservation deposits than the pycnodontids, turtles or ichthyosaurs (which are all rather rare). In Nusplingen, these holes are known from most ammonite groups including perisphinctids (60–80% bitten), aspidoceratids (rarely with that fracture, sometimes conchs are completely destroyed), as well as oppeliids (60–80% bitten) (unpublished field data from GS). In some layers, most ammonite conchs actually show this fracture. In Nusplingen, pycnodontids are extremely rare, while in the Solnhofen–Eichstätt region, pycnodontids are occasionally found but not very common. In Nusplingen, findings are limited to one large *Gyrodus*. Conceivably, the very pointed upper jaw of belemnoids (Klug et al. 2010b, 2020) such as *Acanthoteuthis* or *Hibolithes* was capable of puncturing the shell of smaller ammonites, which permitted them to eventually produce a larger hole to pull out the soft parts (Fig. 7). Remarkably, none of the nautilid conchs known from Nusplingen were broken in this way; this may be linked either with the thicker and thus more resistant conch of the nautilids, with their lower abundance or with a different habitat or behaviour.

## Conclusions

We describe ammonite soft parts from the early Tithonian of the Solnhofen-Eichstätt Archipelago. These soft parts are remarkable since they appear to be in their nearly original arrangement although they are not in the conch anymore. This more or less original arrangement facilitates homologization of organs in spite of the taphonomic loss of many fine anatomical details. We could identify most parts of the digestive tract and, although with a lower degree of support, of the mantle cavity with gills, the reproductive tract, the central nervous system with eye capsules, and the hyponome. Presuming our interpretations are correct, this would suggest that the overall arrangement of soft parts do not differ fundamentally from the arrangement from other cephalopods such as modern *Nautilus*. The discovery of probably male reproductive organs in this *Subplanites* supports the hypothesis that microconch ammonites were the males.

We discuss the possible reason for this isolated occurrence. Circumstantial evidence allows two alternative hypothetical explanations: (1) one option is that the ammonite had died and started to decay, whereby the soft tissue became loose and, maybe due to wave action, the soft parts slipped out of the conch. The second option (2) is that a predator such as a coleoid cephalopod (belemnoid or octobrachian coleoid), a pycnodont fish or a turtle attacked the ammonite, cracked the conch near the rear end of the body chamber thereby loosening the soft body from its main attachment, and pulled it out of the conch. For an unknown reason, it dropped the soft parts and did not retrieve it. Possibly, the arm crown ripped off in this process.

## Data Availability

All specimens illustrated and described are stored at the Staatliches Museum für Naturkunde in Stuttgart, Germany, and at the Bayerische Staatssammlung für Paläontologie und Geologie in Munich, Germany.
